# Apatinib inhibits glioma cell malignancy in patient-derived orthotopic xenograft mouse model by targeting thrombospondin 1/myosin heavy chain 9 axis

**DOI:** 10.1038/s41419-021-04225-2

**Published:** 2021-10-11

**Authors:** Hui Yao, Jiangang Liu, Chi Zhang, Yunxiang Shao, Xuetao Li, Zhengquan Yu, Yulun Huang

**Affiliations:** 1 Department of Neurosurgery, the First Affiliated Hospital of Soochow University, No188, Shizi Street, Suzhou, 215007 Jiangsu China; 2 Department of Neurosurgery, Dushu Lake Hospital Affiliated of Soochow University, Suzhou, 215124 Jiangsu China

**Keywords:** Drug development, Cell invasion, Pharmacodynamics, Proteomics, Transcriptomics

## Abstract

We determined the antitumor mechanism of apatinib in glioma using a patient-derived orthotopic xenograft (PDOX) glioma mouse model and glioblastoma (GBM) cell lines. The PDOX mouse model was established using tumor tissues from two glioma patients via single-cell injections. Sixteen mice were successfully modeled and randomly divided into two equal groups (*n* = 8/group): apatinib and normal control. Survival analysis and in vivo imaging was performed to determine the effect of apatinib on glioma proliferation in vivo. Candidate genes in GBM cells that may be affected by apatinib treatment were screened using RNA-sequencing coupled with quantitative mass spectrometry, data mining of The Cancer Genome Atlas, and Chinese Glioma Genome Atlas databases, and immunohistochemistry analysis of clinical high-grade glioma pathology samples. Quantitative reverse transcription-polymerase chain reaction (qPCR), western blotting, and co-immunoprecipitation (co-IP) were performed to assess gene expression and the apatinib-mediated effect on glioma cell malignancy. Apatinib inhibited the proliferation and malignancy of glioma cells in vivo and in vitro. Thrombospondin 1 (THBS1) was identified as a potential target of apatinib that lead to inhibited glioma cell proliferation. Apatinib-mediated THBS1 downregulation in glioma cells was confirmed by qPCR and western blotting. Co-IP and mass spectrometry analysis revealed that THBS1 could interact with myosin heavy chain 9 (MYH9) in glioma cells. Simultaneous THBS1 overexpression and MYH9 knockdown suppressed glioma cell invasion and migration. These data suggest that apatinib targets THBS1 in glioma cells, potentially via MYH9, to inhibit glioma cell malignancy and may provide novel targets for glioma therapy.

## Introduction

Glioblastomas (GBMs) are the most common type of malignant primary brain tumor, astrocytomas, and GBMs together account for approximately 75% of all gliomas [1]. The prognosis of GBM is dismal, with a median overall survival (OS) rate of only 12–18 months for newly diagnosed GBM patients [2, 3].

Temozolomide and antiangiogenic drugs are first-line chemotherapeutics for GBM [4, 5], and bevacizumab is the most common anti-vascular endothelial growth factor (VEGF) drug [5]. Apatinib (YN968D1) is an oral small molecule antitumor drug that inhibits VEGF-induced tyrosine phosphorylation of VEGF receptor 2 and activation of its downstream pathways [6, 7]. Notably, apatinib showed better therapeutic potential than other antiangiogenic agents such as ramoximab, bevacizumab, and sunitinib [8], and it is used as a second-line therapeutic agent for most solid tumors [9, 10]. Because of its effective anti-vascular properties [11, 12], apatinib has been widely used in chemotherapy for patients with advanced cancer [13–15], leading to significant improvement in the survival rates of several cancers including advanced gastric cancer, advanced non-squamous non-small cell lung cancer (NSCLC), and metastatic breast cancer [16, 17]. Importantly, apatinib has also been shown to have antitumor effects on gliomas from clinical trials and in vitro experiments [18, 19] and has also been used in patients with recurrent glioma or those undergoing advanced glioma treatments with promising therapeutic effects [20–23]. Several clinical trials of apatinib and temozolomide combination therapy of recurrent GBM showed promising results in tumor regression [24, 25]. Moreover, minimal adverse effects have been noted following apatinib administration in patients.

Therefore, this study was designed to elucidate the underlying antitumor mechanism of action of apatinib on glioma cells. A patient-derived orthotopic xenograft (PDOX) glioma mouse model was established and two human GBM cell lines from patients (N14042 and N14069, Supplementary Table S1), as well as one commercially available cell line (U87), were used to identify potential target genes of apatinib. The mechanisms of action of the target genes were determined through RNA-sequencing (RNA-seq), proteomics, bioinformatics, immunohistochemistry (IHC), and molecular biology techniques such as quantitative polymerase chain reaction (qPCR) and western blot analysis. These findings provide novel insights into the mechanism of action of apatinib and propose thrombospondin 1 (THBS1) as a potential target in GBM therapy.

## Results

### Apatinib inhibits the progression of primary glioma cells in vivo

**Fig. 1:**
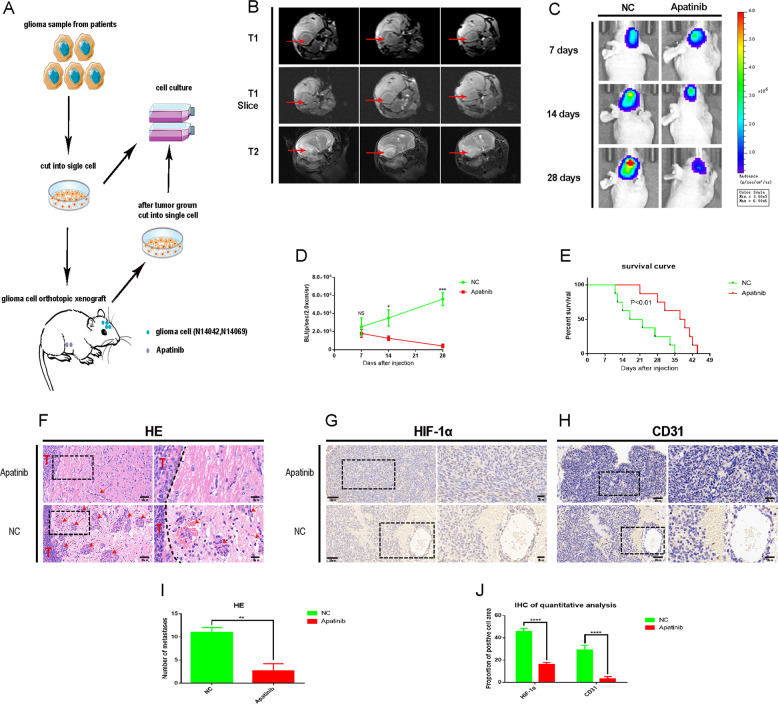
Apatinib inhibits the progression of glioma cells in vivo. **A** Schematic diagram of patient-derived orthotopic xenograft (PDOX) model establishment. **B** Detection of mouse tumorigenesis by 3.0T nuclear magnetic resonance (NMR). **C** Representative images of bioluminescence in mice on days seven, 14, and 28 after cell implantation. **D** Quantitative analysis of bioluminescence images for the apatinib and control treatment groups. Data are shown as the mean ± standard deviation (SD), *n* = 3, ***p < 0.001, *p < 0.05, NS > 0.05, compared with the control, Student’s *t* test. **E** The overall survival (OS) of mice in the apatinib and control treatment groups. Data are shown as the mean ± standard deviation (SD), *n* = 8, ***p < 0.001, *p < 0.05, NS > 0.05, compared with the control, Student’s *t* test. **F** Hematoxylin & eosin (H&E) staining of mouse brain sections differences showed between apatinib and NC group. *T* represents the tumor, the dotted line represents the boundary between the tumor and normal brain tissue, and red arrows indicate metastases. Representative immunohistochemistry (IHC) staining images of **G** CD31, and **H** HIF-1α in tumor sections of apatinib and normal control (NC) groups. **I** Quantitative analysis of H&E staining images regarding the number of metastases in the apatinib and negative control groups. Data are shown as mean ± standard deviation (SD), *n* = 3, ^#^*p* = NS, *p < 0.05, **p < 0.01, ***p < 0.001, ****p < 0.0001, Student’s *t* test. **J** Quantitative analysis of the IHC results concerning the proportion of CD31 and HIF-1α-positive cells in the apatinib and negative control groups. Data are shown as the mean ± SD, *n* = 3, **p < 0.01 compared with the control, analysis of variance (ANOVA) test.

The PDOX model was successfully established (Fig. 1A) as evidenced by magnetic resonance imaging (MRI; Fig. 1B) and it was used to evaluate the clinical efficacy of apatinib. Bioluminescence imaging and hematoxylin and eosin (H&E) staining revealed that tumor progression in the apatinib group was inhibited compared with the control group (Fig. 1C, F). In addition, apatinib inhibited the invasion of glioma cells as evidenced by the H&E staining (Fig. 1F, I). IHC quantitative analysis showed that apatinib inhibited tumor angiogenesis and hypoxia (Fig. 1G, H, J). Importantly, this could contribute to the improved survival evidenced by the significantly longer OS times for the apatinib group than the control group in the survival analysis (Fig. 1E; *p* < 0.01). Taken together, the results suggest that apatinib inhibited glioma cell progression in vivo.

### Apatinib inhibits proliferation, migration, and invasion of primary glioma cells in vitro

**Fig. 2:**
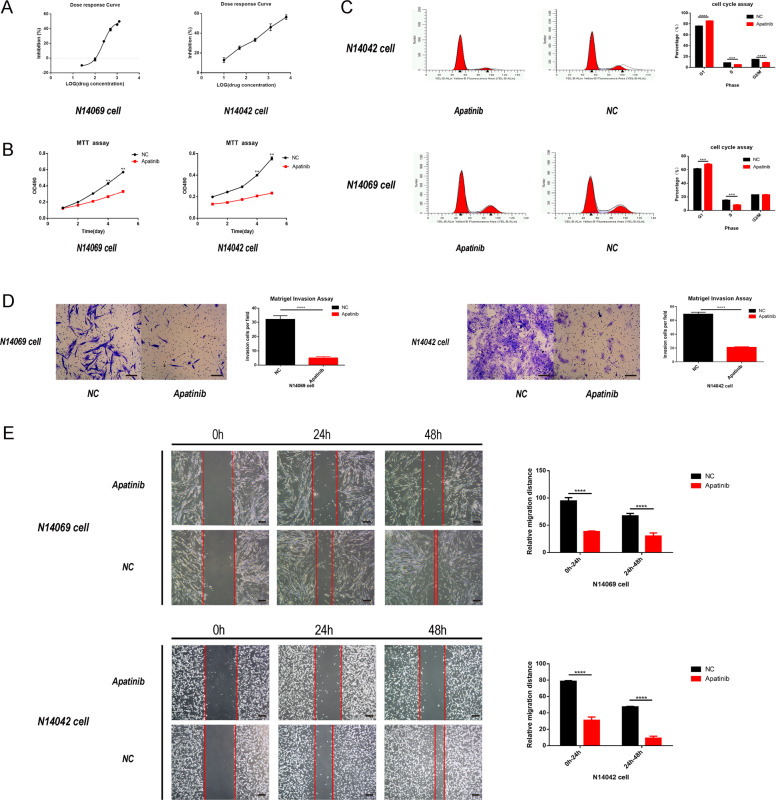
Apatinib inhibits the proliferation and invasion of glioma cells in vitro. **A** Half-maximum inhibitory concentration (IC_50_) of two human glioma cell lines: N14042 cells (292.9 µmmol/L) and N14069 cells (231.6 µmol/L). **B** Representative proliferation curves showed that proliferation of glioma cells was significantly inhibited by apatinib treatment (at IC_50_). **C** Representative flow cytometry profiles and corresponding histograms showed that the cell cycle of glioma cells was significantly blocked by apatinib treatment (at IC_50_). **D**, **E** Representative images and corresponding histograms of glioma cell numbers and diameters showed that the invasion and migration ability of glioma cells was significantly inhibited by apatinib treatment (concentration within 5% of the lethal dose; **E**: ×40 magnification, **D**: ×100 magnification). Data are shown as mean ± standard deviation (SD), *n* = 3, *p < 0.05, **p < 0.01, ***p < 0.001, ****p < 0.0001, Student’s *t* test.

Next, the effects of apatinib on glioma cell proliferation, migration, and invasion in vitro were examined. After confirming that apatinib could inhibit the proliferation of glioma cells (Fig. 2A), N14042 and N14069 human GBM cells were treated with apatinib at their half-maximum inhibitory concentration (IC_50_) values of 292.9 µmol/L and 231.6 µmol/L, respectively. Cell cycle detection analysis was performed at the 36 h time point following apatinib treatment. Glioma cell proliferation was inhibited in the apatinib group (Fig. 2B; *p* < 0.05 vs. control group). The propidium iodide-flow cytometry (PI-FCM) analysis revealed that glioma cells in the apatinib group arrested at the G1 phase, suggesting that apatinib blocked cell cycle progression (Fig. 2C; *p* < 0.05 vs. control group). In addition, a reduced capacity for migration and invasion was observed in N14069 and N14042 cells treated with apatinib (Fig. 2D, E; *p* < 0.05 vs. control group).

### Apatinib inhibits glioma cells by targeting the THBS1 gene

**Fig. 3:**
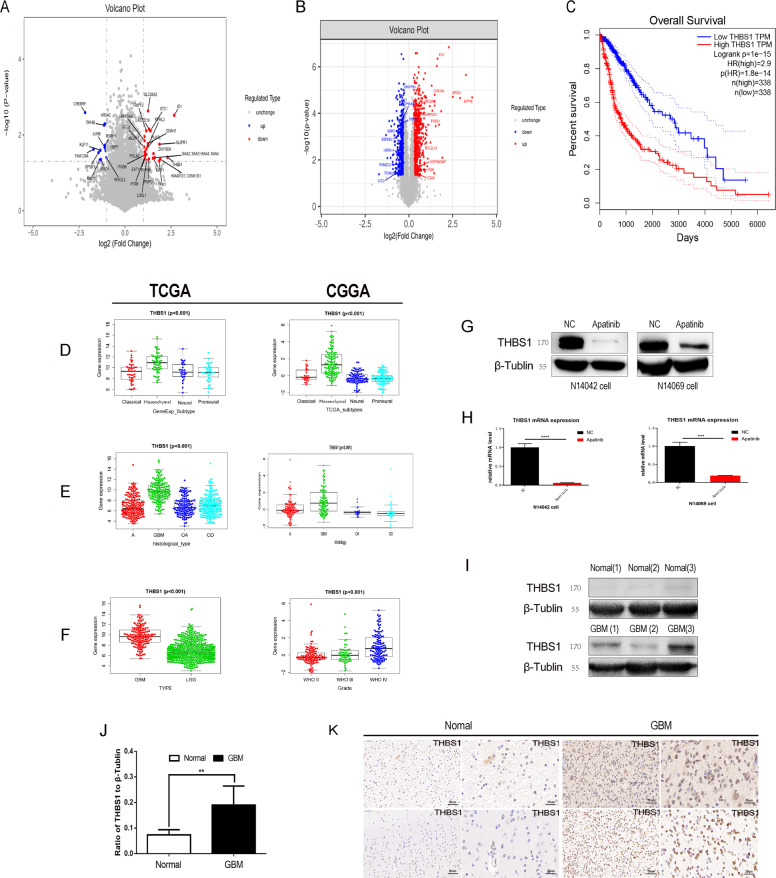
Apatinib may inhibit the expression of thrombospondin 1 (THBS1) in gliomas. *THBS1* mRNA is inversely correlated with overall survival (OS) in patients with glioma and relates to glioma histology and the glioblastoma (GBM) subtype. **A** Volcano plot of differentially expressed genes in N14042 and N14069 glioma cells after apatinib treatment (1vs1). The downregulated genes common in both cell lines were analyzed. **B** Volcano plot of differentially expressed proteins in the two cell lines that have been mixed and processed (3vs3). The downregulated proteins were analyzed and overlapped with the RNA-seq data. **C** The Cancer Genome Atlas (TCGA) equation data sets were used for survival analysis for high-level/low-level THBS1 expression in glioma. **D** The expression features in GBM subtypes were examined using the TCGA and the Chinese Glioma Genome Atlas (CGGA) data sets. **E**, **F** TCGA-seq and CGGA-seq data sets were used to estimate the correlation between *THBS1* mRNA expression and tumor grade according to the World Health Organization (WHO). **E** mRNA levels of different histological subtypes were examined: astrocytoma (A), oligoastrocytoma (OA), oligodendroglioma (OD), and GBM, with CGGA-seq and TCGA-seq data sets. **G**, **H** Protein and mRNA expression levels after apatinib treatment were detected by western blot and qPCR analyses. Data are shown as mean ± standard deviation (SD), *n* = 3, ^#^*p* = NS, *p < 0.05, **p < 0.01, ***p < 0.001, ****p < 0.0001, Student’s *t* test. **I**, **J** Protein expression levels of THBS1 in normal and tumor tissues were detected by western blotting. Data are shown as mean ± SD, *n* = 5, ^#^*p* = NS, *p < 0.05, **p < 0.01, ***p < 0.001, ****p < 0.0001, Student’s *t* test. **K** Immunohistochemistry (IHC) staining in normal and tumor tissues.

Next, the gene targets of apatinib were screened using RNA-seq (N14042 cell line, 1vs1; N14069 cell line, 1vs1) and proteomics (N14069+N14042 cell mixture, 3vs3) to better understand its mechanism of action. RNA-seq revealed that apatinib downregulated 380 genes in N14069 cells (Supp. Fig. S1A) and 649 genes in N14042 cells (Supp. Fig. S1B), among which 24 genes were common to both cell lines (Fig. 3A). Quantitative mass spectrometry on the mixture of N14069 and N14042 GBM cells was also performed to identify differentially expressed proteins, which revealed 559 downregulated proteins in the apatinib group (Fig. 3B and Supp. Fig. S1C). Comparative analysis of the RNA-seq and quantitative mass spectrometry results led to the identification of nine genes with altered expression in the apatinib treatment group (Supp. Table S2). As the *THBS1* gene was the only downregulated gene in common between the RNA-seq and quantitative mass spectrometry results (Supp. Table S2), its role as a gene target of apatinib in glioma cells was further examined. A total of 692 TCGA sequencing (TCGA-seq) samples and 301 CGGA sequencing (CGGA-seq) samples were evaluated to explore the relationship between *THBS1* mRNA expression and subtype, histology type, and OS of glioma patients. *THBS1* mRNA expression in mesenchymal gliomas was higher compared with preneural gliomas, classic gliomas, and neurogliomas in both the TCGA and CGGA databases (Fig. 3D). *THBS1* showed the highest expression in GBM in both TCGA and CGGA databases (Fig. 3E, F), and low *THBS1* expression was associated with higher OS rates (Fig. 3C). qPCR and western blotting revealed that *THBS1* mRNA and THBS1 protein expression in glioma cells treated with apatinib was lower compared with the control group (Fig. 3G, H). Furthermore, THBS1 showed higher expression in tumor tissues compared with normal tissues (Fig. 3I–K). These results suggest that *THBS1* is a probable target of the inhibitory effect of apatinib on glioma cells and that THBS1 expression may be associated with GBM.

### The role of the THBS1 gene in glioma cells

**Fig. 4:**
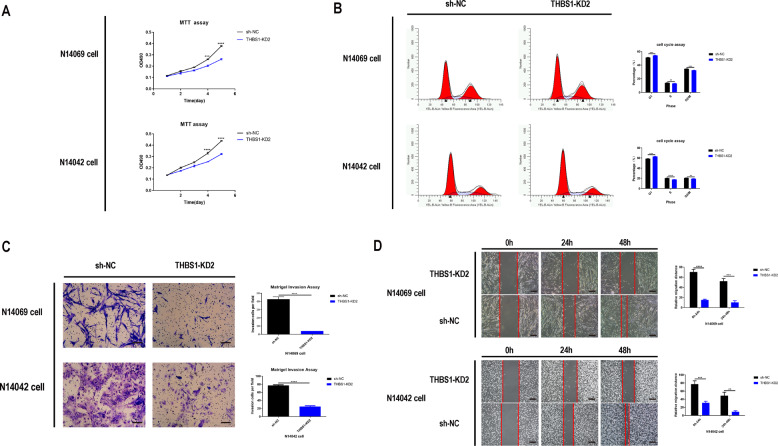
Knockdown of thrombospondin 1 (THBS1) inhibits the proliferation, migration, and invasion of glioma cells. **A** Representative proliferation curves showed that glioma cell proliferation was significantly inhibited following THBS1 knockdown. **B** Representative flow cytometry profiles and corresponding histograms showed that THBS1 downregulation led to cell cycle arrest of glioma cells at the G1 phase. Representative images and corresponding histograms of **C** Transwell migration (×100 magnification) and **D** scratch assay (×40 magnification) results showed that THBS1 downregulation significantly inhibited the migration and invasion ability of glioma cells, respectively. Data are shown as mean ± standard deviation (SD), *n* = 3, *p < 0.05, **p < 0.01, ***p < 0.001, ****p < 0.0001, Student’s *t* test.

**Fig. 5:**
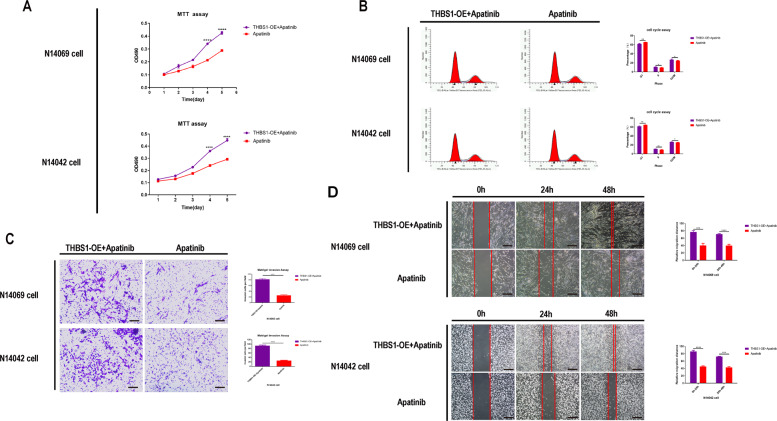
Thrombospondin 1 overexpression (THBS1-OE) can counteract the inhibitory effect of apatinib on glioma cell proliferation, migration, and invasion. **A** Representative proliferation curves showed that glioma cell proliferation was significantly inhibited upon apatinib treatment (at IC_50_) compared with the THBS1-OE plus apatinib treatment group. **B** Representative flow cytometry profiles and corresponding histograms showed that apatinib treatment compared with THBS1-OE plus apatinib treatment led to cell cycle arrest of glioma cells (at IC_50_). Representative images and corresponding histograms of **C** Transwell migration (×100 magnification) and **D** scratch assay results (×40 magnification) showing the migration and invasion of glioma cells, respectively. Compared with THBS1-OE plus apatinib-treated cells, apatinib-treated cells inhibited the migration and invasion of glioma cells (apatinib concentration is within 5% of the lethal dose). Data are shown as mean ± standard deviation (SD), *n* = 3, *p < 0.05, **p < 0.01, ***p < 0.001, ****p < 0.0001, Student’s *t* test.

The expression, transfection, and knockdown efficiency of *THBS1* gene fragments in N14069, N14042, and U87 glioma cells were detected by fluorescence microscopy and qPCR, respectively (Supp. Fig. S2A–D). The shTHBS1 knockdown 2 (KD2), which had the highest knockdown efficiency, was used for subsequent experiments (Supp. Fig. S2E). Similar to treatment with apatinib, the migration, and invasion of glioma cells was inhibited after knockdown of the THBS1 gene (Fig. 4A–D). Next, it was determined whether THBS1 overexpression could reverse the inhibitory effect of apatinib on glioma cell, and a THBS1 overexpression lentiviral construct was transfected into glioma cells. The expression and transfection efficiency was detected by fluorescence microscopy and western blot (Supp. Figs. S2F, 6F, 6G). Compared with THBS1 overexpression (THBS1-OE) plus apatinb, apatinib inhibited glioma cell growth and progression (Fig. 5A–D). The results revealed that [1]: apatinib can inhibit the malignant growth of gliomas; [2] apatinib downregulated *THBS1* gene expression, and *THBS1* gene downregulation inhibited malignant glioma growth; and [3] THBS1-OE can counteract the inhibitory effect of apatinib. Taken together, the results suggest that apatinib can inhibit the proliferation and invasion of glioma cells by regulating the THBS1 gene.

### THBS1 can interact with MYH9

**Fig. 6:**
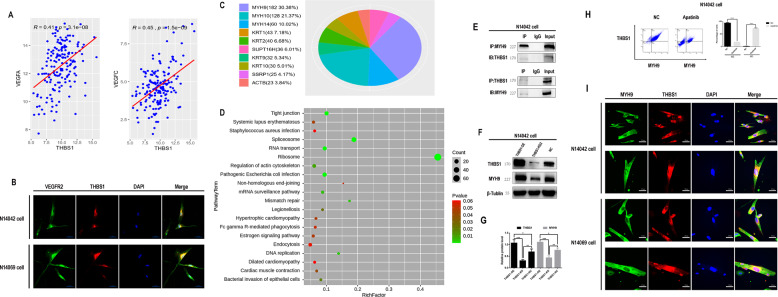
Interaction between thrombospondin 1 (THBS1) and myosin heavy chain 9 (MYH9)/vascular endothelial growth factor receptor 2 (VEGFR2) in glioma cells. **A** Analysis of The Cancer Genome Atlas (TCGA) equation THBS1 and VEGFR2 co-expression data sets. **B** Immunofluorescence images of THBS1 and VEGFR2 colocalization in glioma cells. The nuclei were stained with DAPI. Images were captured using a laser confocal microscope. **C** Immunoprecipitation followed by mass spectrometry showed that the MYH9 peptide showed the highest sequence coverage. **D** Pathway analysis for MYH9 revealed that the tight junction pathway showed the highest enrichment. **E** Immunoprecipitation followed by western blot analysis revealed the interaction between THBS1 and MYH9 in glioma cells. **F**, **G** Western blot analysis showed that MYH9 protein expression decreased when THBS1 was knocked down. **H** Representative flow double-staining showed that the expression of THBS1–MYH9 was inhibited after the administration of apatinib. **I** Immunofluorescent images of THBS1 and MYH9 expression in glioma cells. The nuclei were stained with DAPI. Images were captured by a laser confocal microscope. Data are shown as mean ± standard deviation (SD), *n* = 3, ^#^*p* = NS, *p < 0.05, **p < 0.01, ***p < 0.001, ****p < 0.0001, Student’s *t* test.

Therefore, THBS1 is the target gene of apatinib regarding the inhibition of glioma growth. It is well known that apatinib is a tyrosine kinase inhibitor (TKI) drug, which inhibits the expression of VEGFR2. THBS1 is a large multidomain protein that interacts with many proteins [26], and can modulate tumor progression and metastasis [27]. THBS1 has been reported to interact directly or indirectly with VEGFR2 and can interfere with angiogenesis [28, 29]. Our analysis of the TCGA database suggested that THBS1, VEGFRA, and VEGFRC are co-expressed genes in glioma samples (Fig. 6A). Moreover, immunofluorescence showed cytoplasmic and cytomembrane colocalization of VEGFR2 and THBS1 (Fig. 6B). These results indicate that apatinib can inhibit the VEGFR2/THBS1 interaction and that apatinib might be a multi-target inhibitor. Therefore, immunoprecipitation followed by mass spectrometry was performed to identify potential binding partners of THBS1. The MYH9 protein showed the highest coverage for the THBS1 binding peptide (Fig. 6C). KEGG pathway analysis revealed that MYH9 was enriched in the tight junction pathway (Fig. 6D). Co-immunoprecipitation (co-IP) showed that THBS1 interacted with MYH9 (Fig. 6E), and THBS1 downregulation led to MYH9 downregulation (Fig. 6F, G). By the way, the flow double-staining experiment showed that apatinib could inhibit the expression of THBS1–MYH9 (Fig. 6H). Immunofluorescence revealed cytoplasmic colocalization of MYH9 and THBS1 (Fig. 6I).

### Apatinib affects MYH9 by regulating THBS1

**Fig. 7:**
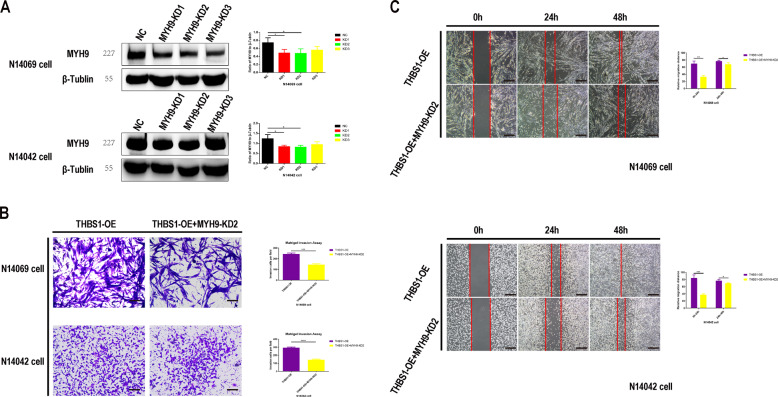
Myosin heavy chain 9 (MYH9) knockdown inhibits glioma cell invasion and migration in cells that overexpress thrombospondin 1 (THBS1). **A** Western blot images and corresponding histograms of MYH9 protein in glioma cells transient transfected with three MYH9 short hairpin RNA (shMYH9): KD1, KD2, and KD3. Representative images and corresponding histograms of **B** Transwell migration (×100 magnification) and **C** scratch assay results (×40 magnification) showing the migration and invasion of glioma cells, respectively. Compared with THBS1-OE only, in cells that overexpress THBS1 and were transiently transfected with shMYH9, the glioma cells showed inhibited migration and invasion. Data are shown as mean ± standard deviation (SD), *n* = 3, *p < 0.05, **p < 0.01, ***p < 0.001, ****p < 0.0001, Student’s *t* test.

Three specific constructs to knockdown MYH9 expression (KD1, KD2, and KD3) were designed and transfected into glioma cells that stably overexpressed THBS1 to determine the role of MYH9 in glioma cell migration and invasion. Western blot analysis revealed that shMYH9 KD2 showed greater knockdown efficiency compared with KD1 and KD3 (Fig. 7A). MYH9 knockdown inhibited glioma cell migration and invasion, even when THBS1 was overexpressed (Fig. 7B, C), indicating that the effect of apatinib on glioma cells likely involves the THBS1/MYH9 axis.

### Apatinib inhibited malignancy of glioma cells in PDOX model

**Fig. 8:**
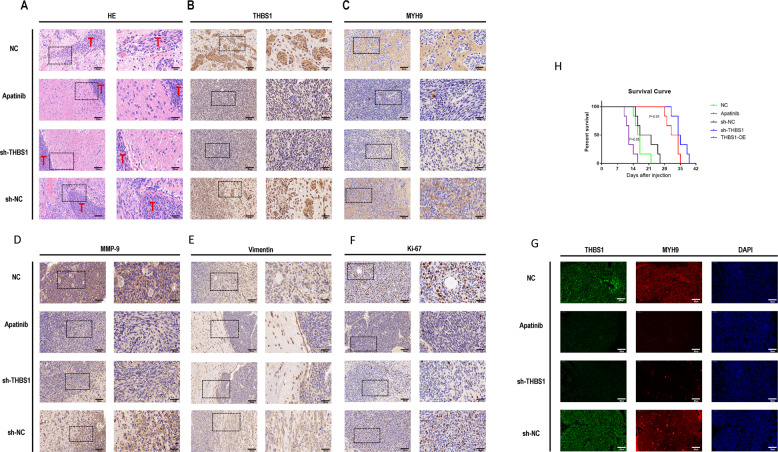
Apatinib inhibited the proliferation and invasion of glioma cells through the THBS1/MYH9 axis in vivo. Representative four groups (apatinib, normal control [NC], shTHBS1, and negative virus control [sh-NC]) in **A** hematoxylin and eosin (H&E) staining and immunohistochemistry (IHC) staining images of **B** THBS1, **C** MYH9, **D** Ki-67, **E** matrix metalloprotease 9 (MMP-9), and **F** vimentin of tumor sections. **G** Immunofluorescence images of mouse brain tumor tissue showed the same results as the IHC images of THBS1 and MYH9. **H** Survival analysis showing the overall survival (OS) of mice in the THBS1-OE, apatinib, NC, shTHBS1, and sh-NC groups. Data are shown as the mean ± standard deviation (SD), *n* = 8; **p < 0.01 compared with the control, analysis of variance (ANOVA) test.

In vivo, lower malignancy was noted of glioma cells in the apatinib compared with the normal control (NC) group, and lower malignancy was also found in the shTHBS1 group compared with the negative virus control (sh-NC) group by H&E staining and survival curve analysis (Fig. 8A, H). IHC of mouse brain slices showed that THBS1, MYH9, Ki-67, matrix metalloproteinase-9 (MMP-9), and vimentin expression levels were decreased in the apatinib treatment group compared with the NC group. Similarly, THBS1, MYH9, Ki-67, MMP-9, and vimentin expression levels were decreased in the shTHBS1 group compared with the sh-NC group (Fig. 8B–F). Ki-67, MMP-9, and vimentin are commonly used to evaluate the proliferative and invasive capacities of tumor cells, respectively, where increased expression indicates a higher capacity. Immunofluorescence of mouse brain tumor tissue revealed the same results as IHC of THBS1 and MYH9 (Fig. 8G). This suggests that apatinib can also inhibit the proliferation and invasion of glioma cells in vivo and has a role through the THBS1–MYH9 axis.

## Discussion

Apatinib was found to prolong the OS in patients with recurrent glioma. Literature reports have shown that the mechanism of action of apatinib differs in vitro based on the type of tumor [30–32]. As the molecular mechanism of apatinib has not been characterized in glioma cases, its antitumor effect and in vitro mechanism were determined using RNA-seq and quantitative mass spectrometry. Apatinib was shown to inhibit glioma cell malignancy in vivo using a PDOX mouse model, as well as in vitro using cell lines, and the *THBS1* gene was identified as the probable target of apatinib. *THBS1* was the only common gene with an altered expression between primary glioma cell lines. Moreover, analysis of the TCGA and CGGA databases revealed that THBS1 expression was significantly different between high-grade and low-grade gliomas. Furthermore, THBS1 expression was significantly different between normal brain tissues and those from glioma patients. Notably, THBS1 downregulation or decreased expression resulted in decreased glioma cell invasion and migration, and increased survival of glioma patients, respectively.

The potential involvement of the THBS1/MYH9 axis on apatinib-mediated inhibition of glioma cell migration and invasion was demonstrated in previous reports. Immunoprecipitation and mass spectrometry revealed MYH9 as a potential binding partner of THBS1 in glioma cells and suggested that the latter may regulate the function of the MYH9 protein. MYH9 knockdown experiments confirmed the association between MYH9 and glioma progression. These findings provide new insights for the use of apatinib in postoperative chemotherapy of recurrent gliomas and present a novel strategy for the treatment of recurrent gliomas that involves targeting the THBS1/MYH9 axis.

THBS1 is a member of the multidomain and multi-functional calcium-binding extracellular glycoprotein family. The family consists of five protein-coding genes: *THBS1*, thrombospondin 2 (TSP-2), TSP-3, TSP-4, and TSP-5 (ref. [33]). THBS1 was initially identified as an endogenous protein inhibitor of angiogenesis, which inhibits endothelial cell migration and neovascularization [34, 35]. However, recent studies have found that THBS1 is highly expressed in certain malignant tumors and can promote angiogenesis [36, 37]. Similarly, with regard to tumor cell invasion, the function of THBS1 is complex and controversial [38–40], and may vary by cell type, tissue type, and subcellular localization [41]. In the current study, THBS1 was highly expressed in gliomas and could promote the proliferation and invasion of glioma cells. Apatinib was shown to potentially target THBS1 in gliomas resulting in the inhibition of the capacity of glioma cells to proliferate and invade.

THBS1 promotes physiological and pathological processes by regulating the activity, availability, and structure of ligands, and ultimately regulates the response of cells to environmental stimuli in a context-dependent manner [42]. In this study, immunoprecipitation led to the discovery of MYH9 as a likely binding partner of THBS1 in glioma cells. Although most THBS1 interactions occur in the extracellular space, in the extracellular matrix, or near the plasma membrane [42, 43], some studies have shown that THBS1 interactions can also occur within cells. Consistent with previous studies, THBS1 and MYH9 were found by immunofluorescence to colocalize around the nucleus, and an intracellular interaction between THBS1 and MYH9 was observed from the co-IP data [42, 44, 45].

The MYH9 gene encodes the heavy chain of class II non-muscle myosin type A protein, which is expressed in all eukaryotic cells. MYH9 is involved in various processes that require force generation and actin cytoskeletal rearrangements, such as cell migration, adhesion, cell shape maintenance, and signal transduction [46]. In recent years, studies have found that MYH9, as a protein involved in cytoskeletal reorganization, pseudopodia formation, and migration [47], plays an important role in the progression of glioma [48] and other solid tumors [49], and MYH9 acts as an oncogene in almost all solid and hematological tumors [50]. At present, targeted glioma therapy research is an area of avid interest [51, 52], and different targets may represent novel therapeutic strategies. Elucidation of the THBS1/MYH9 axis in regard to the inhibitory effect of apitinib on glioma malignancy may provide new targets for the treatment of gliomas. In future studies, we plan to investigate the role of the apatinib-mediated upregulation of genes in glioma cells, and how apatinib can affect the interaction between VEGFR2 and THBS1.

## Conclusion

**Fig. 9:**
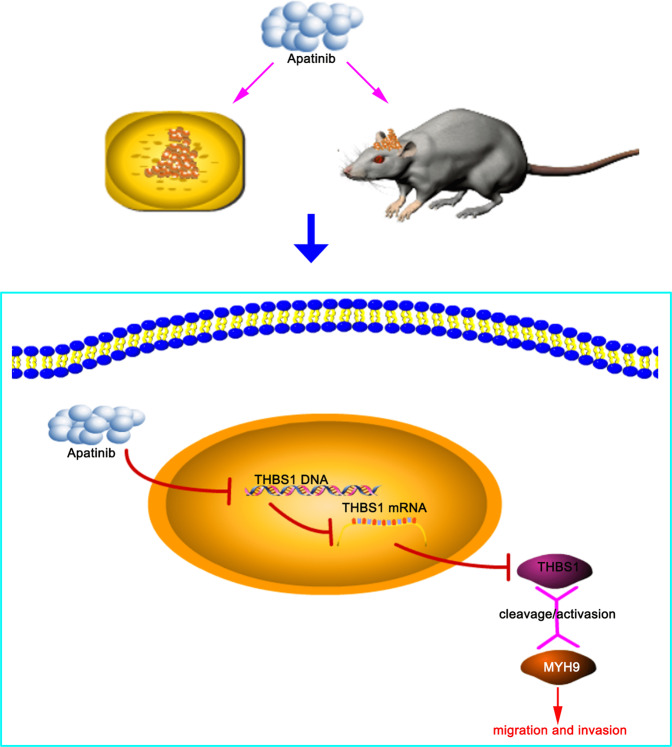
Experimental mechanism diagram. Schematic diagram illustrating apatinib-mediated inhibition of gliomas in vitro and in vivo and apatinib-mediated regulation of the THBS1/MYH9 axis.

These findings provide insights into the underlying mechanism of action of apatinib on glioma proliferation, migration, and invasion via the THBS1/MYH9 axis and could contribute to improvement in glioma therapy (Fig. 9). In addition, these data may propose new research directions regarding targeted glioma therapeutic drug development against THBS1 and the THBS1/MYH9 axis.

## Materials and methods

### Animals and grouping

Sixteen female nude mice (4–5 weeks old, 20 g) were housed in a sterile environment, following a 12:12 h-light/dark cycle at 23 °C. Food and water were provided ad libitum. The mice were randomly divided into two equal groups, control or treatment with apatinib (*n* = 8/group). All animal experiments were approved by the Ethics Committee of the First Affiliated Hospital of Soochow University, China.

### PDOX model

Primary glioma cells (1 × 10^7^ cells) obtained from two GBM patients admitted at the First Affiliated Hospital of Soochow University (Supp. Table S1) were passaged for 50 generations before being transfected with lentiviral luciferase (Genechem) and stereotaxically inoculated into mice to establish the intracranial PDOX glioma model [53]. All human experiments were approved by the Ethics Committee of the First Affiliated Hospital of Soochow University, China. On the seventh day after glioma cell inoculation, the mice that survived were intraperitoneally injected with apatinib (200 mg/kg/day) in phosphate-buffered saline (PBS) and saline solution, 4 days/week for 4 weeks [54]. The fresh drug was used for all doses for both in vitro and in vivo survival experiments. MRI (3.0T) was performed to assess whether the tumor was well-formed in mice on day 14 after cell inoculation. Bioluminescence imaging using the in vivo spectral real-time imaging system (IVIS; Blandford, USA) was performed to assess the size of intracranial tumors on days 7, 14, and 28. Triplicate measurements were obtained. After the last bioluminescence imaging on day 28, all mice were continually treated with apatinib, 4 days/week for four weeks, to observe whether they exhibited normal behavior, cachexia, and emotional changes, as well as to assess the difference in survival duration between the treatment and control groups.

### Cell culture

Two primary human GBM cell lines (N14042 and N14069) obtained from two GBM patients as well as the commercial U87 cell line (Shanghai Academy of Biological Sciences) were cultured in a DMEM medium. The two primary GBM cell lines were identified by short tandem repeat DNA profiling (Supp. material 2).

### MTT assay

N14042 and N14069 cells in the logarithmic growth phase were subjected to tryptic digestion, resuspended into a cell suspension, and plated in a 96-well plate (4000 cells/well; apatinib vs. control; knockdown vs. control; overexpression vs. control). Apatinib was added to the cell cultures the following day. Four hours prior to arresting cell growth, 20 µL of the MTT solution (5 mg/mL; Thermo Scientific) was added to each well. After 4 h, the culture medium was removed, and dimethylsulfoxide (100 µL) was added into each well, and plates were placed on an oscillator for 3–5 min to dissolve the formazan particles. The optical density (OD) value was then detected using a microplate reader (Thermo Scientific) at 490/570 nm according to the manufacturer’s instructions.

### Propidium iodide-flow cytometry assay

The PI-FCM assay was performed to determine the effect of apatinib on cell cycle progression in the experimental groups 36 h after drug treatment. When the cells had reached ~80% confluence (N14042 and N14069 cells did not enter the exponential growth phase), tryptic digestion was performed, and cells were resuspended and collected in centrifuge tubes (5 mL). Triplicate wells were prepared for each group (number of cells/well ≥106). The cells were centrifuged at 1300 rpm for 5 min and rinsed with precooled D-Hanks Balanced Salt Solution (D-HBSS; pH7.2–7.4 at 4 °C). Next, the cells were centrifuged at 1300 rpm for 5 min and incubated with precooled ethanol (75% at 4 °C) for at least 1 h, before another centrifugation at 1300 rpm for 5 min. Cells were rinsed with D-HBSS and then stained with the cell staining solution [0.6–1 mL; 40× PI solution (2 mg/mL), 100× RNase solution (10 mg/mL), and 1× D-HBSS at 25:10:1000] for 15 min before being subjected to flow cytometry analysis using an Attune NxT flow cytometer (Thermo Scientific).

### Transwell invasion assay

The matrigel transwell assay was performed to assess glioma cell invasion. Serum-free culture media (500 µL) was added to the upper chambers, and culture media containing 10% FBS was added to the lower chambers of a 24-well Transwell plate. The Matrigel matrix was rehydrated in a 37 °C incubator for 2 h (Corning Costar). N14042 and N14069 cell suspensions (10^5^/insert) were inoculated in both upper (500 μL) after removal of the serum-free culture solutions. The non-invasive cells in the upper chamber were gently removed with a cotton swab. Giemsa staining solution (two to three drops for 3–5 min) were added to the lower surface of the membrane to stain the cells that had invaded. The chamber was soaked and rinsed several times with PBS, then air-dried. Images of each chamber, with three random visual fields of view, were captured using an inverted microscope (Olympus, Japan).

### Scratch assay

A scratch assay was performed to assess the degree of cell migration. N14042 and N14069 cells (5 × 10^5^ cells/well) were inoculated into the six-well plate and cultured overnight at 37 °C under 5% CO_2_ conditions. A 10 µL pipette tip was used to scratch horizontal lines into the monolayer of cells. The cells were carefully rinsed with PBS three times to remove dislodged cells. Serum-free medium was added to the six-well plate and cell culture was continued at 37 °C under 5% CO_2_ conditions. Images were captured at regular time intervals, including 0, 12, and 24 h, using an inverted microscope.

### RNA-sequencing

Total RNA was isolated from cells using the TRIzol reagent and was used for RNA-seq performed with the Agilent 2100 bioanalyzer system. The RNA was reverse transcribed to cDNA using the GeneChip WT PLUS Kit (Thermo Scientific), and the cDNA was purified, fragmented, and hybridized with the microarray probe. After hybridization, the microarray chip (Genechem) was washed and dyed, and images, as well as raw data, were obtained.

### Quantitative mass spectrometry

The proteome was solubilized in sodium dodecyl sulfate (SDS), and then exchanged with urea on a standard filtration device for quantitative mass spectrometry. Peptides (100 μg) eluted from filtration were subsequently labeled using the iTRAQ (isobaric tags for absolute quantitation) labeling kit (AB SCIEX) according to the manufacturer’s instructions. To purify the labeled peptides, the Agilent 1260 infinity II high-pressure liquid chromatography system followed by the nanometer velocity Easy nLC liquid chromatography system (Buffer A: 0.1% formic acid solution; buffer B: 0.1% formic acid + 80% acetonitrile solution) were used. Next, the peptides were passed through an analytical column (equilibrated with 100% Buffer A; Acclaim PepMap RSLC 50 µm × 15 cm, nanoviper, P/N164943; Thermo Scientific, USA) at a flow rate of 300 nL/min of Buffer A. After chromatographic separation, the Q Exactive Plus mass spectrometer (Thermo Scientific) was used for quantitative mass spectrometry analysis. Triplicate measurements were obtained. The selection criteria of fold change >2 and *p* < 0.05 were applied to obtain the top differentially expressed genes or proteins.

### Lentiviral shRNA transfection

THBS1 short hairpin RNA (shTHBS1) knockdown (KD) and THBS1 overexpression lentivirus constructs were inserted into hU6-CMV-Puro-GFP(Gv493) and Ubi-CMV-3FLAG-CBh-gcGFP-Puro (Gv492) vectors (Genechem, China), respectively. The shTHBS1 and THBS1 overexpression lentiviral constructs were transfected into N14069, N14042, and U87cells using the Infection Enhancer P solution (Genechem) according to the manufacturer’s instructions. Three specific myosin heavy chain 9 (MYH9) shRNA (shMYR9) knockdown constructs were also designed (KD1, KD2, and KD3) to determine the role of MYH9 in glioma cell migration and invasion (Supp. Table 3). These three shMYR9 constructs were transfected into N14042 and N14069 glioma cells that stably overexpressed THBS1. Stably transfected cell clones were selected with puromycin.

### qPCR

Total RNA was isolated from N14042 and N14069 cells using the TRIzol kit (Pufei Biotech, China) and reverse transcribed into cDNA (2 μg total RNA per sample) using the M-MLV Reverse Transcriptase kit (Promega) according to the manufacturer’s instructions. Supplementary Table 4 contains the primer sequences used for the reaction. qPCR was performed using the SYBR® Select Master Mix and mRNA expression levels were quantified using the 2^−∆∆Ct^ method.

### Western blotting

Total protein was extracted from N14042 and N14069 cells using radioimmunoprecipitation assay (RIPA) lysis buffer (Beyotime, China). Protein concentration was determined using a bicinchoninic acid (BCA) assay kit (Beyotime, China). Proteins were separated by SDS electrophoresis and separated bands were transferred onto a polyvinylidene fluoride membrane. The membrane was incubated with primary antibody (Supp. Table 5) at 4 °C overnight, followed by horseradish peroxidase (HRP)-conjugated secondary antibody (Supp. Table 5) at room temperature for 1 h. The bands were visualized using an enhanced chemiluminescent (ECL) reagent (Beyotime, China) and ImageJ software (NIH, USA) was used for densitometry analysis.

### IHC and H&E staining

Mice brains were fixed in 4% paraformaldehyde and then embedded in paraffin for IHC and H&E staining. The sections were deparaffinized in xylene and rehydrated in graded ethanol. Endogenous peroxidase activity was quenched using 0.3% hydrogen peroxide, and antigen retrieval was performed by heating to 37 °C. Goat serum (5%) was used to block nonspecific proteins. The mouse brain tumor tissue sections (25 µm) were subjected to immunostaining using primary antibodies (1:100 dilution; Supp. Table 5) at 4 °C overnight followed by incubation with biotin-conjugated secondary antibodies (1:100 dilution) at 37 °C for 1 h. Subsequently, the sections were incubated with avidin–biotin complex peroxidase, stained with diaminobenzidine, and counterstained with H&E. Images were captured using an inverted microscope.

### Immunofluorescence

N14042 and N14069 cells were plated on glass slides in a 12-well plate, cultured overnight, and fixed with 4% paraformaldehyde the following day. Triton X-100 (5%) was used to permeabilize the cells. Cells were then incubated in a 5% bovine serum albumin solution at 37 °C for 30 min. Next, the cells were incubated with primary antibodies (1:100 dilution; Supp. Table 5) at 4 °C overnight, followed by incubation with secondary antibodies (1:100 dilution; Supp. Table 5) at room temperature for 1 h. Staining with 4′,6-diamidino-2-phenylindole was used to identify cell nuclei. The glass slide was visualized under a Perkin Elmer Ultra VIEW VOX fluorescence confocal microscope. Images from 16 random fields of view at ×800 magnification were captured and analyzed.

### Co-IP

Protein was extracted from N14042 cells and protein concentration was quantified using the BCA assay kit (Beyotime, China). Protein (200 µL) was added to magnetic beads (50 µL) pre-rinsed with washing buffer and incubated with primary antibody (1:20 dilution), and incubated at 4 °C overnight with shaking. The mixture was centrifuged at 6.25 × *g* for 5 min at room temperature, the supernatant was discarded, and the magnetic beads were retained. Loading buffer (4×) was subsequently added to the magnetic beads and they were heated at 95 °C for 15 min before centrifugation at 1200 × *g* for 5 min at room temperature. The magnetic beads were discarded, and the supernatant was separated by SDS gel electrophoresis. Separated protein bands were transferred onto a polyvinylidene fluoride membrane and incubated with a secondary antibody (1:100 dilution) at 4 °C overnight. The membrane was then incubated with an antibody specific for the secondary antibody at room temperature for 1 h. Bands were visualized using an ECL reagent and analyzed using ImageJ software (NIH, USA).

### Bioinformatics

Differentially expressed genes or proteins of the apatinib and control groups with corrected *p* values of <0.05 and a fold change of >2.0 were considered significantly differentially expressed. All downregulated genes were compared with all downregulated proteins to find the same gene/protein. The Cancer Genome Atlas (TCGA), Chinese Glioma Genome Atlas (CGGA) database, and Kyoto Encyclopedia of Genes and Genomes (KEGG) were used for analysis.

### Statistical analysis

Data from at least three experimental replicates were presented as the mean ± standard deviation. Statistical analysis was performed using GraphPad Prism v6 (GraphPad software, USA) and comparisons between two groups were performed using Student’s *t* test. Univariate analysis of variance was used to evaluate the differences between more than two groups. Univariate survival analysis for OS rates was performed using the Kaplan–Meier estimator. A *p* value <0.05 was considered statistically significant.

## Supplementary information

Supplementary Figure

Supplementary Figure S1

Supplementary Figure S2

Supplementary materials

## Data Availability

The data that support the findings of this study are openly available in 10.6084/m9.figshare.14965704.
