# Supplementary material for: Apatinib inhibits glioma cell malignancy in patient-derived orthotopic xenograft mouse model by targeting thrombospondin 1/myosin heavy chain 9 axis
Source: Cell Death Dis. 2021 Oct 11;12(10):927. doi: 10.1038/s41419-021-04225-2 (PMC8505401; doi:10.1038/s41419-021-04225-2)
Supplement: Supplementary file 1 — Supplementary Figure [file 41419_2021_4225_MOESM1_ESM.docx]

**Supplementary Figure legends**

**Supplementary Figure S1. RNA sequencing (RNA-seq) and proteomics analyses.** Scatter plots comparing RNA-seq results between apatinib and control groups in (A) N14069 and (B) N14042 cell lines (1vs1). Cells were treated with apatinib at IC_50_ concentrations. A total of 380 and 649 genes were downregulated in N14069 and N14042 cell lines, respectively. The scatter plot shows the distribution of signal intensity between the experimental group and the control group on the rectangular coordinate plane. The abscissa represents the NC group and the ordinate represents the DRUG (apatinib) group. The abscissa and ordinate of each point in the graph represent the signal strength of a probe group in the NC group and apatinib group; the parallel solid green line is the difference reference line. The points in the interval of the reference line represent the probe group with no significant change, the red points outside the interval represent the probe group with relative downregulation in the NC group, and the green points represent the probe group with relative downregulation in the apatinib group. (C) Heatmap showing top 20 differentially expressed proteins in apatinib and control groups (3vs3). Triplicates A1, A2 and A3, and B1, B2, B3, for control and apatinib groups, respectively.

**Supplementary Figure S2. Assessment of thrombospondin 1 (THBS1) short hairpin (sh)RNA (shTHBS1) lentivirus (KD1, KD2, KD3) transfection efficiency.** (A) Assessment of transfection efficiency of shTHBS1 lentivirus in glioma cells at 100× magnification. (B-D) Quantitative polymerase chain reaction (qPCR) histograms of *THBS1* mRNA expression levels after shTHBS1 lentiviral transfection. Data are shown as mean ± standard deviation (SD), n = 3, ^#^p = NS, *p < 0.05, **p < 0.01, ***p < 0.001, ****p < 0.0001, Student’s *t*-test. (E) Assessment of transfection efficiency of shTHBS1-KD2 lentivirus in glioma cells at 100× magnification. Images were captured using a laser confocal microscope. (F) Assessment of transfection efficiency of the shTHBS1-OE lentivirus in glioma cells at 100× magnification. Images were captured using a laser confocal microscope.
