# Supplementary figures and images for: Apatinib inhibits glioma cell malignancy in patient-derived orthotopic xenograft mouse model by targeting thrombospondin 1/myosin heavy chain 9 axis
Source: Cell Death Dis. 2021 Oct 11;12(10):927. doi: 10.1038/s41419-021-04225-2 (PMC8505401; doi:10.1038/s41419-021-04225-2)

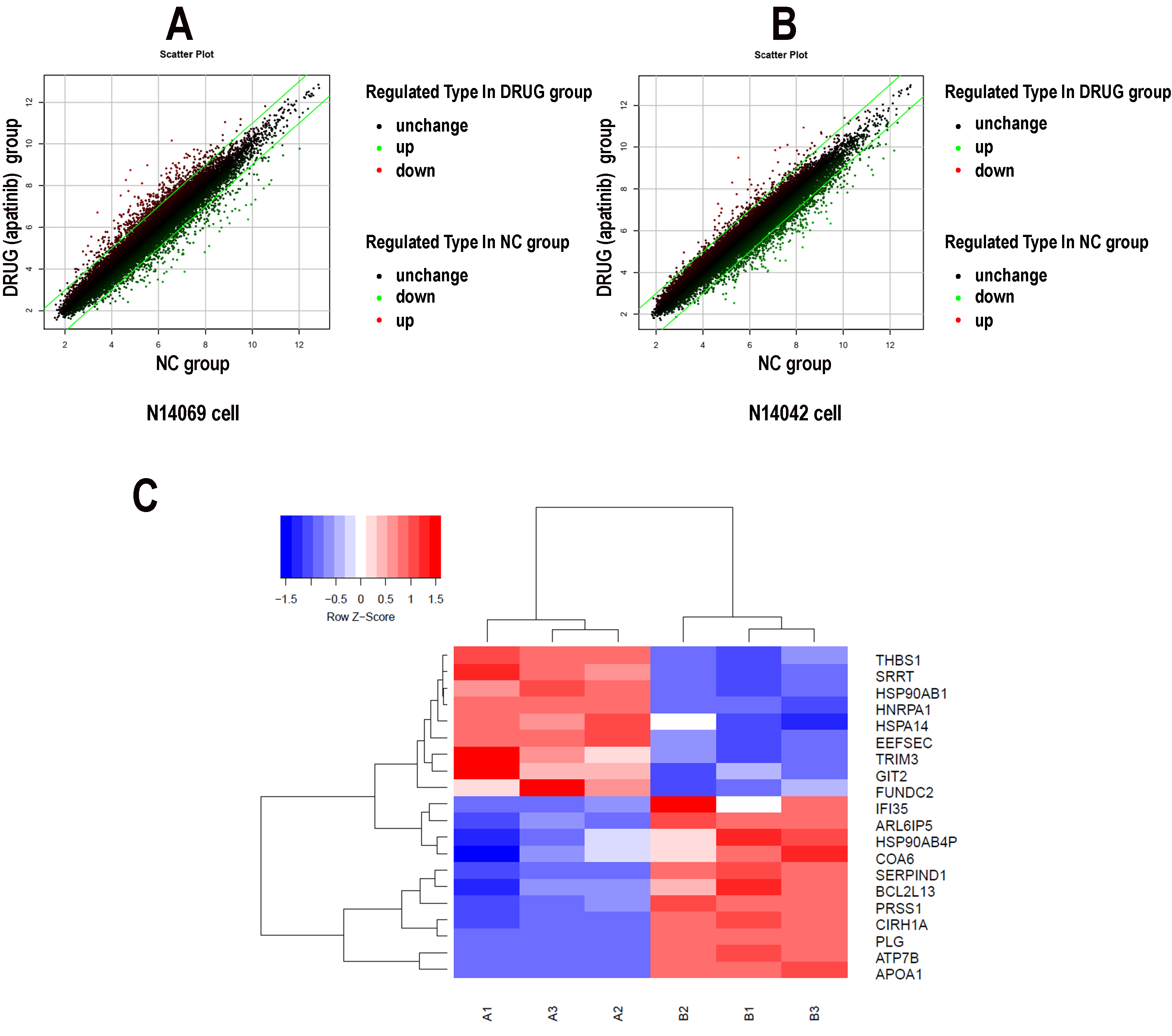

Supplement: Supplementary file 2 — Supplementary Figure S1 [file 41419_2021_4225_MOESM2_ESM.png]

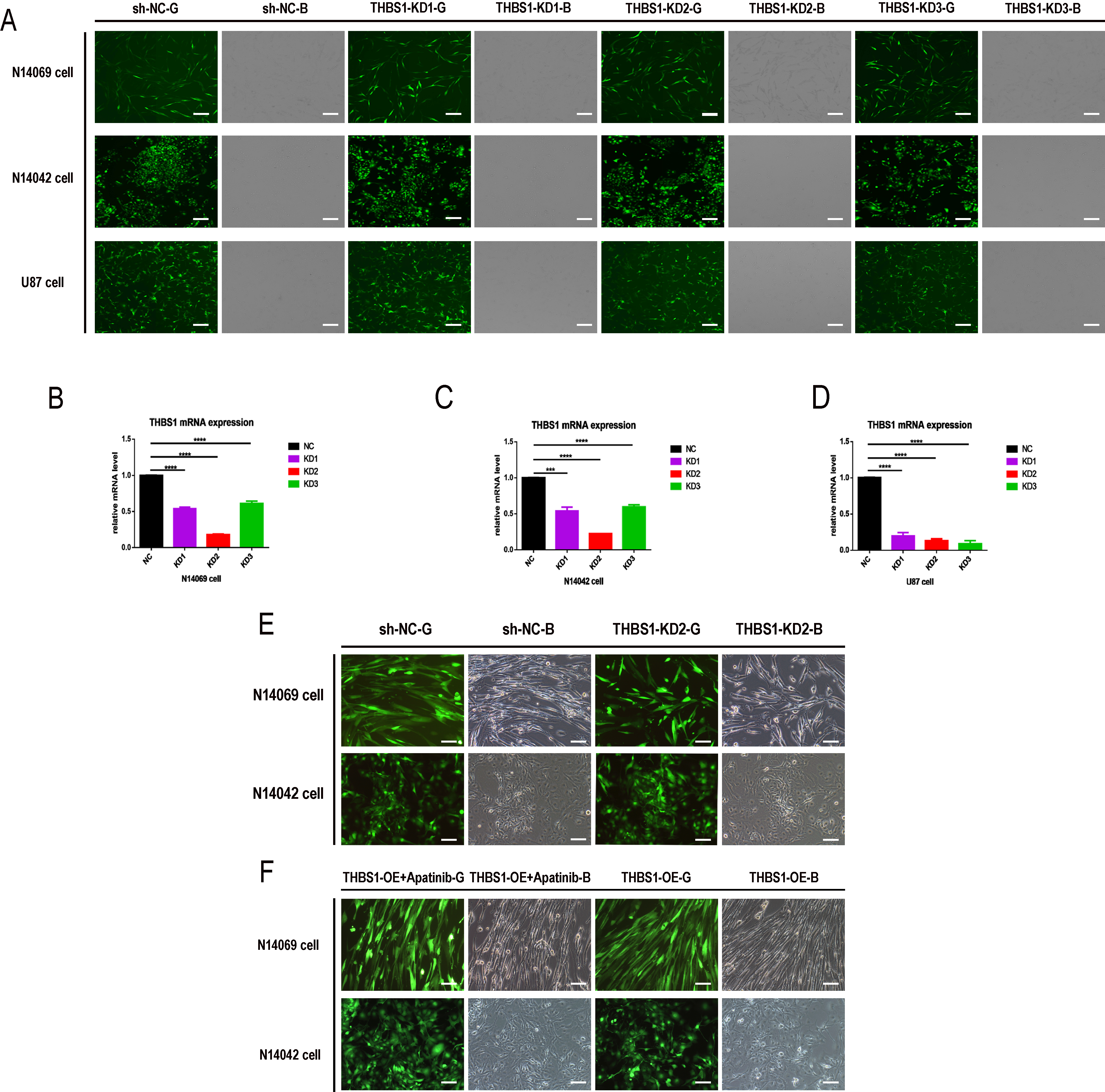

Supplement: Supplementary file 3 — Supplementary Figure S2 [file 41419_2021_4225_MOESM3_ESM.png]
