# Supplementary material for: Apatinib inhibits glioma cell malignancy in patient-derived orthotopic xenograft mouse model by targeting thrombospondin 1/myosin heavy chain 9 axis
Source: Cell Death Dis. 2021 Oct 11;12(10):927. doi: 10.1038/s41419-021-04225-2 (PMC8505401; doi:10.1038/s41419-021-04225-2)

**Supplementary Table 1**. Details regarding the two patients admitted to the First Affiliated Hospital of Soochow University from whom glioma primary cells were obtained.

| **Source of Sample** | **Sex** | **Age** | **Glioma WHO Grade** | **Recurrence** | **Chemotherapy after surgery** | **Overall Survival (months)** | **Histopathology** |
| --- | --- | --- | --- | --- | --- | --- | --- |
| N14069 | Male | 74 | III | Yes | Yes | 13 | Anaplastic astrocytoma |
| N14042 | Male | 56 | IV | Yes | Yes | 10 | GBM |

WHO, World Health Organization; GBM, glioblastoma.

**Supplementary Table 2**. RNA sequencing (RNA-seq) and quantitative mass spectrometric (QMS) analysis of downregulated genes and proteins. A selection criteria of fold change cutoff > 2 and p < 0.05 were applied. THBS1 is the only gene in common between the two analytical methods.

| Downregulated gene (RNA-seq)  (fold change > 2) | Downregulated protein (QMS)  (fold change > 2) |
| --- | --- |
| *CCL2* | THBS1 |
| *THBS1* | PPP1R10 |
| *TNFRSF11B* | TNS3 |
| *XRCC2* | MKI67 |
| *FAM111B* | LDHA |
| *NDC80* | PBK |
| *ID1* | TMPO |
| *HIST1H1B* | RCC2 |
| *COL1A2* | PRC1 |
| *IL7R* | G6PD |
| *DPP4* |  |
| *SGK1* |  |
| *KIAA0101; CSNK1G1* |  |
| *MMP3* |  |
| *HIST1H2BM* |  |
| *NCAPG* |  |
| *CHN1* |  |
| *RRM2* |  |
| *ANKRD30B* |  |

**Supplementary Table 3**. shRNAs or RNA sequences used in the study.

| Gene | Target Seq (5' to 3') | Source |
| --- | --- | --- |
| THBS1 (KD1) | CTATGCTATCACAACGGAGTT | Genechem |
| THBS1 (KD2) | CGTGACTGTAAGATTGTAAAT | Genechem |
| THBS1 (KD3) | CTCTCAAGAAATGGTGTTCTT | Genechem |
| MYH9 (KD1) | GCCGUACAACAAAUACCGCUUTT AAGCGGUAUUUGUUGUACGGCTT | Sangon Biotech |
| MYH9 (KD2) | GCCAAGCUCAAGAACAAGCAUTT AUGCUUGUUCUUGAGCUUGGCTT | Sangon Biotech |
| MYH9 (KD3) | GCCGUACAACAAAUACCGCUUTT AAGCGGUAUUUGUUGUACGGCTT | Sangon Biotech |

shRNA, short hairpin RNA; TSP-1, thrombospondin 1; MYH9, myosin heavy chain 9.

**Supplementary Table 4**. qPCR primers.

| Gene | Forward (5’-3’) | Reverse (5’-3’) |
| --- | --- | --- |
| THBS1 | CTCTCAAGAAATGGTGTTCTT | TCACCACGTTGTTGTCAAGGG |
| GAPDH | TGACTTCAACAGCGACACCCA | CACCCTGTTGCTGTAGCCAAA |

qPCR, quantitative polymerase chain reaction; TSP-1, thrombospondin 1; GAPDH, glyceraldehyde 3-phosphate dehydrogenase.

**Supplemental Table 5**. Antibody information and conditions for use.

| Antibody | Catalogue | Producer | Application |
| --- | --- | --- | --- |
| THBS1 (1) | 37879S | Cell Signaling | WB, 1:1000  IP, 1:100 |
| THBS1 (2) | ab1823 | ABcam | IF, 1:500 |
| MYH9 (1) | 60233-1-Ig | Proteintech | IF, 1:500 |
| MYH9 (2) | 11128-1-AP | Proteintech | WB, 1:3000  IP, 2 ug |
| HIF-1α | ab216842 | ABcam | IHC, 1:325 |
| CD31 | ab28364 | ABcam | IHC, 1:325 |
| VEGFR2 | ab39378 | ABcam | IF,1:500 |
| MMP-9 | 13667S | Cell Signaling | IHC, 1:325 |
| Vimentin | CST#5741 | Cell Signaling | IHC,1:325 |
| Ki-67 | CST#9449S | Cell Signaling | IHC, 1:500 |
| FLAG | CST#8146S | Cell Signaling | IP, 1:50 |
| β-Tubulin | CST#2146S | Cell Signaling | WB, 1:1000 |
| Anti-mouse IgG | CST#7076 | Cell Signaling | WB, 1:2000 |
| Anti-rabbit IgG | CST#7074 | Cell Signaling | WB, 1:2000 |
| Alexa Fluor® Plus 488 | A32723 | Invitrogen | IF, 1:500 |
| Alexa Fluor® Plus 488 | GB25303 | Servicebio | IF,1:300 |
| Alexa Fluor® Plus 594 | A32740 | Invitrogen | IF, 1:500 |
| Alexa Fluor® Plus 594 | GB21303 | Servicebio | IF,1:300 |

TSP-1, thrombospondin 1; MYH9, myosin heavy chain 9; MMP-9, matrix metalloproteinase 9; WB, western blotting; IP, immunoprecipitation; IHC, immunohistochemistry.

**Supplementary material 1**. Thrombospondin 1 (THBS1) overexpression coding sequence.

TGGCCGTTTTTGGCTTTTTTGTTAGACGAAGCTTGGGCTGCAGGTCGACTCTAGAGGATCCCGCCACCATGGGGCTGGCCTGGGGACTAGGCGTCCTGTTCCTGATGCATGTGTGTGGCACCAACCGCATTCCAGAGTCTGGCGGAGACAACAGCGTGTTTGACATCTTTGAACTCACCGGGGCCGCCCGCAAGGGGTCTGGGCGCCGACTGGTGAAGGGCCCCGACCCTTCCAGCCCAGCTTTCCGCATCGAGGATGCCAACCTGATCCCCCCTGTGCCTGATGACAAGTTCCAAGACCTGGTGGATGCTGTGCGGGCAGAAAAGGGTTTCCTCCTTCTGGCATCCCTGAGGCAGATGAAGAAGACCCGGGGCACGCTGCTGGCCCTGGAGCGGAAAGACCACTCTGGCCAGGTCTTCAGCGTGGTGTCCAATGGCAAGGCGGGCACCCTGGACCTCAGCCTGACCGTCCAAGGAAAGCAGCACGTGGTGTCTGTGGAAGAAGCTCTCCTGGCAACCGGCCAGTGGAAGAGCATCACCCTGTTTGTGCAGGAAGACAGGGCCCAGCTGTACATCGACTGTGAAAAGATGGAGAATGCTGAGTTGGACGTCCCCATCCAAAGCGTCTTCACCAGAGACCTGGCCAGCATCGCCAGACTCCGCATCGCAAAGGGGGGCGTCAATGACAATTTCCAGGGGGTGCTGCAGAATGTGAGGTTTGTCTTTGGAACCACACCAGAAGACATCCTCAGGAACAAAGGCTGCTCCAGCTCTACCAGTGTCCTCCTCACCCTTGACAACAACGTGGTGAATGGTTCCAGCCCTGCCATCCGCACTAACTACATTGGCCACAAGACAAAGGACTTGCAAGCCATCTGCGGCATCTCCTGTGATGAGCTGTCCAGCATGGTCCTGGAACTCAGGGGCCTGCGCACCATTGTGACCACGCTGCAGGACAGCATCCGCAAAGTGACTGAAGAGAACAAAGAGTTGGCCAATGAGCTGAGGCGGCCTCCCCTATGCTATCACAACGGAGTTCAGTACAGAAATAACGAGGAATGGACTGTTGATAGCTGCACTGAGTGTCACTGTCAGAACTCAGTTACCATCTGCAAAAAGGTGTCCTGCCCCATCATGCCCTGCTCCAATGCCACAGTTCCTGATGGAGAATGCTGTCCTCGCTGTTGGCCCAGCGACTCTGCGGACGATGGCTGGTCTCCATGGTCCGAGTGGACCTCCTGTTCTACGAGCTGTGGCAATGGAATTCAGCAGCGCGGCCGCTCCTGCGATAGCCTCAACAACCGATGTGAGGGCTCCTCGGTCCAGACACGGACCTGCCACATTCAGGAGTGTGACAAGAGATTTAAACAGGATGGTGGCTGGAGCCACTGGTCCCCGTGGTCATCTTGTTCTGTGACATGTGGTGATGGTGTGATCACAAGGATCCGGCTCTGCAACTCTCCCAGCCCCCAGATGAACGGGAAACCCTGTGAAGGCGAAGCGCGGGAGACCAAAGCCTGCAAGAAAGACGCCTGCCCCATCAATGGAGGCTGGGGTCCTTGGTCACCATGGGACATCTGTTCTGTCACCTGTGGAGGAGGGGTACAGAAACGTAGTCGTCTCTGCAACAACCCCACACCCCAGTTTGGAGGCAAGGACTGCGTTGGTGATGTAACAGAAAACCAGATCTGCAACAAGCAGGACTGTCCAATTGATGGATGCCTGTCCAATCCCTGCTTTGCCGGCGTGAAGTGTACTAGCTACCCTGATGGCAGCTGGAAATGTGGTGCTTGTCCCCCTGGTTACAGTGGAAATGGCATCCAGTGCACAGATGTTGATGAGTGCAAAGAAGTGCCTGATGCCTGCTTCAACCACAATGGAGAGCACCGGTGTGAGAACACGGACCCCGGCTACAACTGCCTGCCCTGCCCCCCACGCTTCACCGGCTCACAGCCCTTCGGCCAGGGTGTCGAACATGCCACGGCCAACAAACAGGTGTGCAAGCCCCGTAACCCCTGCACGGATGGGACCCACGACTGCAACAAGAACGCCAAGTGCAACTACCTGGGCCACTATAGCGACCCCATGTACCGCTGCGAGTGCAAGCCTGGCTACGCTGGCAATGGCATCATCTGCGGGGAGGACACAGACCTGGATGGCTGGCCCAATGAGAACCTGGTGTGCGTGGCCAATGCGACTTACCACTGCAAAAAGGATAATTGCCCCAACCTTCCCAACTCAGGGCAGGAAGACTATGACAAGGATGGAATTGGTGATGCCTGTGATGATGACGATGACAATGATAAAATTCCAGATGACAGGGACAACTGTCCATTCCATTACAACCCAGCTCAGTATGACTATGACAGAGATGATGTGGGAGACCGCTGTGACAACTGTCCCTACAACCACAACCCAGATCAGGCAGACACAGACAACAATGGGGAAGGAGACGCCTGTGCTGCAGACATTGATGGAGACGGTATCCTCAATGAACGGGACAACTGCCAGTACGTCTACAATGTGGACCAGAGAGACACTGATATGGATGGGGTTGGAGATCAGTGTGACAATTGCCCCTTGGAACACAATCCGGATCAGCTGGACTCTGACTCAGACCGCATTGGAGATACCTGTGACAACAATCAGGATATTGATGAAGATGGCCACCAGAACAATCTGGACAACTGTCCCTATGTGCCCAATGCCAACCAGGCTGACCATGACAAAGATGGCAAGGGAGATGCCTGTGACCACGATGATGACAACGATGGCATTCCTGATGACAAGGACAACTGCAGACTCGTGCCCAATCCCGACCAGAAGGACTCTGACGGCGATGGTCGAGGTGATGCCTGCAAAGATGATTTTGACCATGACAGTGTGCCAGACATCGATGACATCTGTCCTGAGAATGTTGACATCAGTGAGACCGATTTCCGCCGATTCCAGATGATTCCTCTGGACCCCAAAGGGACATCCCAAAATGACCCTAACTGGGTTGTACGCCATCAGGGTAAAGAACTCGTCCAGACTGTCAACTGTGATCCTGGACTCGCTGTAGGTTATGATGAGTTTAATGCTGTGGACTTCAGTGGCACCTTCTTCATCAACACCGAAAGGGACGATGACTATGCTGGATTTGTCTTTGGCTACCAGTCCAGCAGCCGCTTTTATGTTGTGATGTGGAAGCAAGTCACCCAGTCCTACTGGGACACCAACCCCACGAGGGCTCAGGGATACTCGGGCCTTTCTGTGAAAGTTGTAAACTCCACCACAGGGCCTGGCGAGCACCTGCGGAACGCCCTGTGGCACACAGGAAACACCCCTGGCCAGGTGCGCACCCTGTGGCATGACCCTCGTCACATAGGCTGGAAAGATTTCACCGCCTACAGATGGCGTCTCAGCCACAGGCCAAAGACGGGTTTCATTAGAGTGGTGATGTATGAAGGGAAGAAAATCATGGCTGACTCAGGACCCATCTATGATAAAACCTATGCTGGTGGTAGACTAGGGTTGTTTGTCTTCTCTCAAGAAATGGTGTTCTTCTCTGACCTGAAATACGAATGTAGAGATCCCGGTATGGACTACAAGGATGACGATGACAAGGATTACAAAGACGACGATGATAAGGACTATAAGGATGATGACGACAAATGAGCTAGCACATAACTTACGGTAAATGGCCCGCCTGGCTGACCGCCCAACGACCCCCGCCCATTGACGTCAATAGTAACGCCAATAGGGACTTTCCATTGACGTCAATGGGTGGAGTATTTACGGTAAACTGCCCACTTGGCAGTACATCAAGTGTATCATATGCCAAGTACG

**Supplemental material 2**. Short tandem repeat (STR) identification atlas of N14069 and N14042 human glioma cells.

N14069 cells

N14042 cells


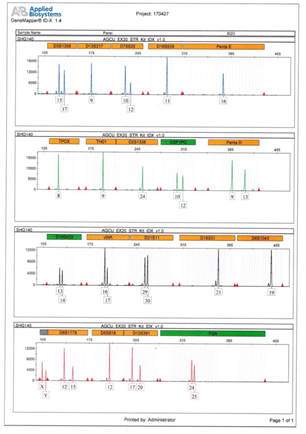

Supplement: Supplementary file 4 — Supplementary materials [file 41419_2021_4225_MOESM4_ESM.doc]
